# A missed case of a large occult intraorbital branch foreign body caused by tree branch laceration: case report

**DOI:** 10.3389/fmed.2026.1827512

**Published:** 2026-06-03

**Authors:** Bolun Xu, Shufeng Xu, Yixuan Tong, Zongduan Zhang

**Affiliations:** 1 Quzhou Affiliated Hospital of Wenzhou Medical University, Quzhou People's Hospital, Quzhou, Zhejiang, China; 2 Eye Hospital and School of Ophthalmology and Optometry, Wenzhou Medical University, Wenzhou, Zhejiang, China

**Keywords:** intraorbital foreign body, magnetic resonance imaging, ocular trauma, orbital cellulitis, vegetative foreign body

## Abstract

**Purpose:**

This study aimed to report a rare case of a large occult intraorbital branch foreign body initially misdiagnosed as orbital cellulitis and conjunctival contusion, and to summarize clinical experience to improve the diagnosis and prognosis of similar cases.

**Methods:**

A retrospective case report following the CARE guidelines, detailing the clinical manifestations, diagnostic process, surgical intervention, and follow-up outcomes of a 60-year-old male patient with a large occult intraorbital branch foreign body.

**Results:**

A 60-year-old male presented with right ocular swelling and pain for 2 days after a tree branch laceration and was initially diagnosed with right orbital cellulitis and conjunctival contusion. Symptoms did not improve after conventional anti-infective treatment. Orbital magnetic resonance imaging (MRI) revealed a linear abnormal signal in the right medial rectus muscle, consistent with an intraorbital foreign body (highly suggestive of a tree branch) with multiple surrounding infectious foci. Emergency surgery was performed to remove a 2 cm branch foreign body, combined with debridement, drainage, and standardized post-operative anti-infective and anti-inflammatory therapy. Visual acuity gradually recovered: right eye visual acuity improved to 0.5 at discharge and 0.8 at the 1-month follow-up. At the 2-month follow-up, diplopia had resolved completely, and the right eye showed full extraocular motility without limitation.

**Conclusion:**

For patients with ocular trauma caused by vegetative foreign bodies, clinicians should maintain a high suspicion for intraorbital foreign bodies, perform detailed history taking and comprehensive physical examinations, and perform orbital MRI in a timely manner to avoid misdiagnosis and missed diagnosis. Computed tomography is the primary screening modality for orbital trauma, while MRI is the preferred examination for suspected vegetative intraorbital foreign bodies. Early surgical removal of the foreign body combined with standardized anti-infective treatment is crucial for improving prognosis and reducing severe complications.

## Introduction

Intraorbital foreign bodies represent a challenging condition in ocular trauma, especially vegetative foreign bodies such as tree branches, bamboo chips, and wood fragments. These lesions are characterized by insidious early symptoms, high risk of infection, and considerable diagnostic difficulty (1, 4). Because vegetative foreign bodies have a density similar to orbital soft tissue, they are easily misdiagnosed as simple orbital cellulitis or conjunctival contusion in the early stage. Delayed diagnosis and treatment may lead to progressive infection, orbital abscess, osteomyelitis, and even intracranial infection, seriously threatening visual function and patient safety (5).

Magnetic resonance imaging (MRI) provides excellent soft-tissue resolution and can clearly demonstrate the location, shape, and adjacent relationships of vegetative foreign bodies within the orbit. Computed tomography (CT) remains the first-line screening tool for general orbital trauma, whereas MRI is the preferred imaging modality for diagnosing intraorbital vegetative foreign bodies (3, 4). The Chinese Expert Consensus on the Diagnosis and Treatment of Intraorbital Foreign Bodies (2021) clearly recommends that patients with ocular trauma and a definite history of vegetative foreign body contact should undergo imaging as early as possible to rule out intraorbital foreign bodies (3). Here, we report a case of a large occult intraorbital branch foreign body initially misdiagnosed as orbital cellulitis and conjunctival contusion. The diagnosis was finally confirmed by orbital MRI after ineffective conventional treatment, and the patient achieved good recovery following surgical intervention. This case aims to raise clinical awareness of occult intraorbital vegetative foreign bodies, standardize the diagnostic workflow, and improve the management of such conditions.

## Case presentation

### Patient history

A 60-year-old male presented to the Ophthalmology Clinic of Quzhou People's Hospital on 3 November 2023 with a 2-day history of right ocular swelling and pain after a tree branch laceration. During physical labor 2 days before admission, he sustained a right ocular scratch by a tree branch, with immediate tearing and foreign body sensation. He self-administered levofloxacin eye drops without obvious relief. Ocular swelling and pain gradually developed and worsened over the following 2 days. His medical history was unremarkable except for prior colon surgery, and he denied any drug allergies. No relevant personal, marital, or family history was noted.

### Initial clinical examination and diagnosis

Ophthalmic examination: uncorrected visual acuity was 0.2 in the right eye and 0.8 in the left eye. Intraocular pressure was 26 mmHg in the right eye and 18 mmHg in the left eye. The right eyelid was swollen; the bulbar conjunctiva was severely congested and edematous. The right eye showed mild proptosis and painful limitation of extraocular movements. The corneas were clear; the anterior chambers were of normal depth; the pupils were round and reactive. Both lenses showed mild cortical opacities. Optic discs, maculae, and retinal vessels were unremarkable.

### Initial diagnosis

1. Right orbital cellulitis
2. Right conjunctival contusion
3. Age-related cataracts (both eyes)

Treatment: intravenous cefuroxime sodium 1.5 g q8h; topical levofloxacin eye drops and tobramycin–dexamethasone eye drops four times daily.

### Re-examination and further diagnostic evaluation

On 6 November 2023, symptoms persisted without improvement.

Re-examination: visual acuity remained 0.2 in the right eye; intraocular pressure was 28 mmHg. The right eye was relatively fixed with limited abduction, elevation, and adduction. The bulbar conjunctiva was markedly congested and edematous, with yellowish-white discharge in the conjunctival sac (Figure 1). Conjunctival perforation was suspected at the 9 o'clock (nasal) position of the right eye.

**Figure 1:**
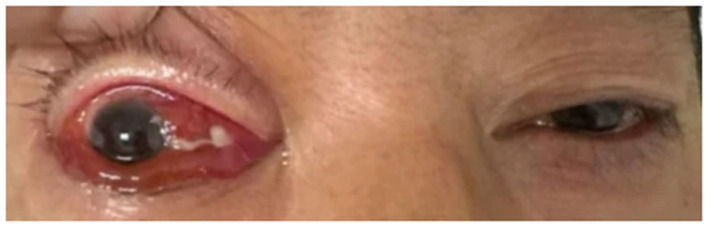
Ocular signs of the patient at the second visit. The right eyelid was swollen, the eyeball was fixed, the bulbar conjunctiva was significantly congested and edematous, and a large amount of yellow-white secretions was visible in the conjunctival sac.

Further history revealed poor lighting at the time of injury; the patient still denied foreign body entry. Orbital MRI was promptly performed. Orbital non-contrast MRI showed significant thickening of the right medial rectus muscle with a linear abnormal signal within the muscle belly, highly suggestive of a tree branch foreign body with multiple surrounding infectious foci (Figure 2).

**Figure 2:**
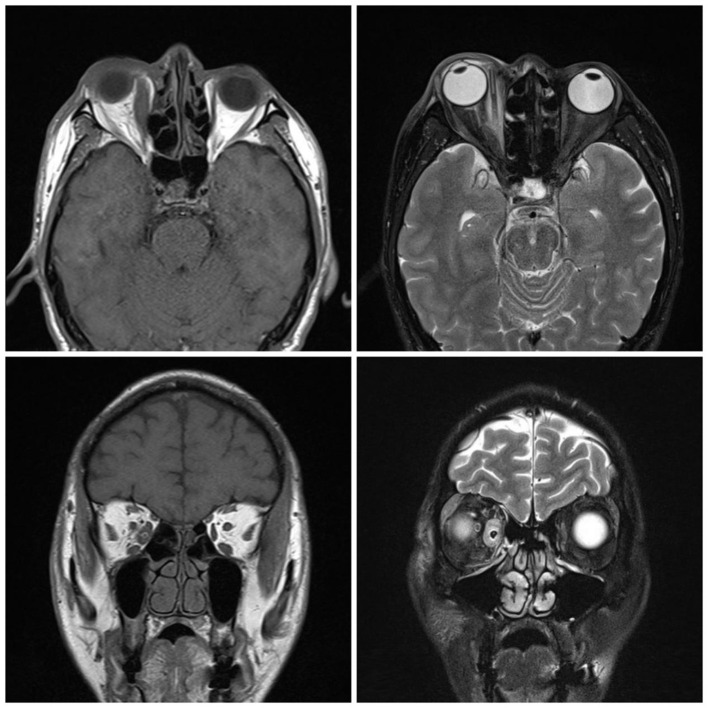
Orbital magnetic resonance imaging findings. The right medial rectus muscle was significantly thickened, with a long strip of high-signal shadow on T1WI and T2WI in the muscle, with clear edges, suggesting an intraorbital foreign body with surrounding inflammation.

### Final diagnosis and hospitalization

#### Final diagnosis

1. Right intraorbital foreign body (vegetative)
2. Right orbital cellulitis
3. Suspected right eyeball perforating injury
4. Age-related cataracts (both eyes)
5. The patient was admitted for emergency surgery on the same day.

### Surgical intervention

Under general anesthesia, emergency right orbital foreign body removal with debridement and suturing was performed. A sinus tract was identified deep to the bulbar conjunctiva at the medial canthus, containing purulent secretions. A branch foreign body approximately 2 cm in length was embedded in the medial rectus muscle belly, 6 mm posterior to its insertion (Figure 3).

**Figure 3:**
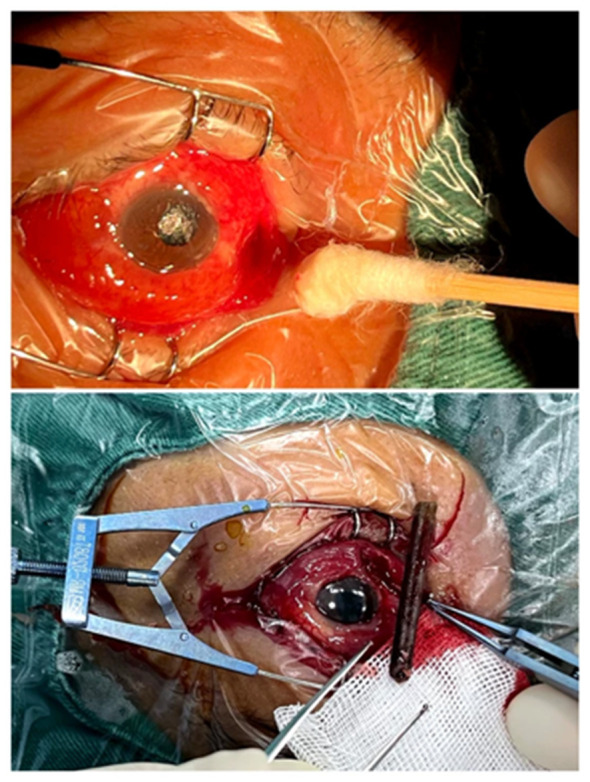
Intraoperative exploration findings. A sinus tract was found at the inner canthus of the right eye, and a tree branch foreign body about 2 cm in length was removed from the muscle belly of the medial rectus muscle during the operation.

After removal of the foreign body, necrotic tissue and purulent debris were thoroughly debrided. The tract was irrigated with povidone –iodine solution, then repeatedly flushed with diluted gentamicin and normal saline. A soft drainage strip was placed to ensure adequate outflow of inflammatory exudate.

### Post-operative treatment and follow-up outcomes

Post-operative treatment included intravenous cefuroxime sodium 1.5 g q8h and dexamethasone 5 mg once daily. Local ocular care was maintained.

The patient was discharged on 13 November 2023. Right eye visual acuity was 0.5; conjunctival congestion and edema had improved; the drainage strip was removed, and no obvious purulent discharge was observed.

1-month follow-up: right eye visual acuity improved to 0.8; the patient reported diplopia and limited adduction. Neurotrophic therapy was prescribed.

2-month follow-up**:** diplopia had resolved completely; visual acuity remained stable at 0.8; the right eye had full extraocular motility in all directions (Figure 4). Orbital inflammation had resolved completely, and ocular structure and function recovered well. The clinical timeline of presentation, diagnosis, treatment, and follow-up is summarized in Table 1.

**Figure 4:**
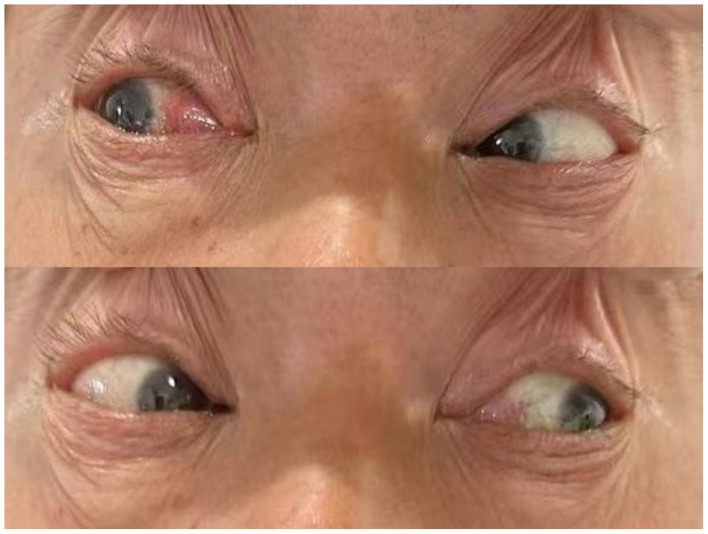
Ocular signs at the 2-month post-operative follow-up. The right eye had no limitation of adduction and other eye movements, the eyelid swelling and conjunctival congestion completely subsided, and the ocular appearance and function recovered well.

**Table 1:** Timeline of clinical presentation, diagnosis, treatment, and follow-up outcomes.

Time point	Clinical events
1 November 2023	The patient suffered a right ocular laceration by a tree branch during labor, with immediate lacrimation and foreign body sensation; self-administered levofloxacin eye drops.
3 November 2023	First visit to the hospital with right ocular swelling and pain for 2 days; initial diagnosis of orbital cellulitis and conjunctival contusion; systemic and local anti-infective treatment initiated.
6 November 2023	Re-examination with unrelieved symptoms, accompanied by eye fixation and limited movement; orbital MRI performed, confirming an intraorbital branch foreign body with infection; admitted to the hospital for emergency surgery.
6 November 2023	Emergency surgery: foreign body removal, debridement, suturing and drainage; post-operative anti-infective and anti-inflammatory treatment.
13 November 2023	Discharged from the hospital with right eye visual acuity recovered to 0.5, relieved conjunctival edema, and no purulent secretions.
6 December 2023 (1-month follow-up)	Right eye visual acuity improved to 0.8 with diplopia and limited adduction; neurotrophic therapy was prescribed.
6 February 2024 (2-month follow-up)	Diplopia disappeared, right eye visual acuity remained stable at 0.8, and there was no limitation of eye movement in all directions; complete recovery of ocular function.

### Diagnostic assessment

#### Differential diagnosis

Initial differential diagnoses included simple right orbital cellulitis, right conjunctival contusion, and early orbital abscess. Clinical signs (eyelid swelling, conjunctival congestion, and edema) were consistent with orbital cellulitis, and the patient denied foreign body entry, leading to the initial misdiagnosis. After ineffective anti-infective treatment with progressive limitation of eye movements, intraorbital foreign body with secondary infection and suspected eyeball perforating injury were added to the differential diagnosis.

#### Imaging and intraoperative confirmation

Orbital MRI was key to the final diagnosis. MRI showed a characteristic linear abnormal signal in the medial rectus muscle, consistent with a vegetative foreign body (tree branch), surrounded by multiple infectious lesions. Intraoperative exploration directly confirmed a 2 cm branch foreign body and an infected sinus tract, representing the diagnostic gold standard.

### Therapeutic intervention

#### Surgical treatment

Emergency surgical removal of the foreign body was the core intervention. The approach was tailored to the foreign body location (medial rectus muscle). Infected tissue and purulent debris were thoroughly debrided, and the sinus tract was copiously irrigated to eradicate infectious foci. Placement of a drainage strip ensured adequate post-operative drainage and prevented recurrent infection.

#### Pharmacological treatment

Systemic anti-infective therapy with cefuroxime sodium was used perioperatively to cover common pathogens associated with orbital vegetative foreign body infections. Post-operative low-dose dexamethasone reduced orbital inflammation and tissue edema. Neurotrophic medications were prescribed during follow-up to support the recovery of extraocular muscle function and relieve diplopia.

## Discussion

We present a 60-year-old male with a large occult intraorbital branch foreign body initially misdiagnosed as orbital cellulitis and conjunctival contusion. Symptoms failed to improve with conventional anti-infective treatment. Orbital MRI confirmed the diagnosis, and emergency surgical removal combined with standardized post-operative therapy resulted in excellent recovery. This case highlights the insidious presentation, high misdiagnosis rate, and significant infection risk of intraorbital vegetative foreign bodies and provides important clinical insights.

### Clinical features and risks of intraorbital vegetative foreign bodies

Most intraorbital vegetative foreign bodies result from accidental trauma, often from tree branches or bamboo fragments (1). Such foreign bodies frequently harbor bacteria and fungi, tend to degrade and fragment in orbital tissue fluid, and create a favorable environment for microbial proliferation, increasing the risk of infection (1). In this case, ocular swelling and pain developed 2 days after injury and worsened despite anti-infective treatment, with elevated intraocular pressure and restricted eye movement. MRI showed multiple infectious foci around the foreign body, consistent with the typical infectious course of vegetative foreign bodies. The infection rate of orbital vegetative foreign bodies is much higher than that of metallic or glass foreign bodies, and most are mixed infections (2). Prolonged retention may lead to orbital abscess, periorbital osteomyelitis, and even intracranial infection, threatening vision and life (6).

### Diagnostic challenges

Early diagnosis is difficult for two main reasons. First, the injury environment and subtle initial signs mimic common conditions such as orbital cellulitis, leading to misdiagnosis (3). Second, CT is the standard first-line screening tool for orbital trauma, but it has low sensitivity for vegetative foreign bodies because their density resembles soft tissue. MRI, with its superior soft-tissue resolution, reliably demonstrates vegetative foreign bodies as characteristic abnormal signals and defines their relationship to extraocular muscles and the optic nerve (3, 4). In this case, imaging was not performed at the first visit, and diagnosis relied only on clinical examination. After treatment failure, a timely orbital MRI clearly identified the foreign body and infectious foci, guiding subsequent surgery.

### Lessons from initial management deficiencies

Despite the favorable final outcome, several deficiencies occurred in the initial management:

1. Insufficient risk assessment: the tree branch injury mechanism was not fully appreciated, and the intraorbital foreign body was not included in the initial differential diagnosis.
2. Delayed imaging: imaging was not performed at the first visit, which was inconsistent with the 2021 Chinese Expert Consensus (3).
3. Incomplete history taking: key details (poor lighting and exact mechanism of injury) were not adequately explored.
4. Delayed re-evaluation: treatment failure did not trigger prompt re-assessment until severe signs appeared.

### Clinical implications

This case provides clear guidance:

1. For any ocular trauma with a history of vegetative foreign body contact, always include intraorbital foreign body in the differential diagnosis, even if the patient denies foreign body entry.
2. Use CT as the first screening tool for orbital trauma; use MRI as the preferred examination for suspected vegetative foreign bodies.
3. Obtain detailed injury history and maintain close follow-up for patients with unclear presentations or poor treatment response.
4. Educate patients to return promptly if swelling, pain, or eye movement abnormalities worsen.

## Conclusion

Early and accurate diagnosis followed by timely surgical removal is the cornerstone of managing intraorbital vegetative foreign bodies. Clinicians must recognize the insidious and high-risk nature of vegetative ocular trauma. CT is the primary screening modality for orbital trauma, while MRI is the preferred examination for detecting and characterizing vegetative intraorbital foreign bodies. A comprehensive approach combining history, clinical examination, and appropriate imaging allows individualized treatment in line with clinical consensus, minimizing ocular damage and improving patient prognosis. Early surgical removal of the foreign body combined with standardized anti-infective and anti-inflammatory therapy is essential to reduce severe complications such as orbital abscess and intracranial infection and to preserve visual function.

## Data Availability

The original contributions presented in the study are included in the article/supplementary material, further inquiries can be directed to the corresponding authors.
